# Cellular tropism exhibited by human T lymphotropic virus type 1 (HTLV-1) and type 2 (HTLV-2)

**DOI:** 10.1186/1742-4690-8-S1-A176

**Published:** 2011-06-06

**Authors:** Priya Kannian, Han Yin, Rami Doueiri, Patrick L Green

**Affiliations:** 1 Center for Retrovirus Research, Department of Veterinary Biosciences, and Comprehensive Cancer Center and Solove Research Institute, The Ohio State University, Columbus, OH, 43210, USA

## Background

HTLV-1 predominantly transforms CD4+T cells in vitro and induces leukemia and neurological disease in vivo, whereas HTLV-2 shows a preference for CD8+T cell transformation in vitro with limited in vivo pathology. To better understand cellular tropism of HTLV-1 and HTLV-2 early after in vivo infection we determined proviral load and gene expression kinetics of these viruses in purified CD4+ and CD8+T cells from newly infected rabbits.

## Materials and methods

New Zealand White rabbits (four/group) were inoculated intravenously with HTLV-1 (Ach), HTLV-2 (pH6neo), and control irradiated producer cells. Blood was collected pre- (week 0) and post-inoculation (weeks 1,2,4,6,8,&12) for detecting antibody responses using line blot assay, and proviral load and viral gene expression in purified CD4+ and CD8+ T cells using real-time PCR.

## Results

HTLV-1 and HTLV-2 infected rabbits seroconverted and had detectable proviral loads in both CD4+ and CD8+T cells by 1 wk post infection. HTLV-1 showed slightly higher CD4+T cell proviral loads early, but overtime the virus was detected at higher levels in CD8+T cells. In general HTLV-2 proviral loads were lower than HTLV-1 and throughout the experimental time course the predominant infected cell was the CD8+T cell. HTLV-1 gene expression levels (gag/pol, tax/rex, and hbz) peaked early in CD4+T cells, but overall expression levels over time were higher in CD8+T cells. HTLV-2 gene expression was detected in both CD4+ and CD8+T cells, but was consistently higher in CD8+T cells throughout the study. Viral determinants of tropism with emphasis on Env will also be discussed.

## Conclusions

In the infected rabbit, HTLV-1 shows an early preference for CD4+T cells, but over the12 wk study the majority of cells harboring and expressing the virus are CD8+T cells. In contrast, in this early stage of infection, HTLV-2 reveals a preference for CD8+T cells. We speculate that this differential tropism between HTLV-1 and HTLV-2 contributes to the distinct pathobiology of these two related viruses.

